# Supplementary material for: Easily Processable, Highly Transparent and Conducting Thiol-Functionalized Reduced Graphene Oxides Langmuir-Blodgett Films
Source: Molecules. 2021 May 4;26(9):2686. doi: 10.3390/molecules26092686 (PMC8124529; doi:10.3390/molecules26092686)
Supplement: Supplementary file 1 [file molecules-26-02686-s001.zip › molecules-1188069-supplementary.pdf]

Supporting Information

S1. Elemental Composition Analysis of mRGO-120 by XPS

The XPS spectra of mRGO-120,-150 and tRGO-180 are shown in **Figure S1**, **S2** and **S3**, respectively. The high resolution C1s XPS spectrum of all resulting materials shows sp2-hybridized carbon as a major component, indicating products are highly reduced even after relatively low reaction temperature (120 °C). The high resolution S2p XPS spectrum of mRGO-120 and -150 clearly shows the binding energy at 164 eV corresponding to C–SH as a major deconvoluted peak, whereas the high resolution S2p XPS spectrum of tRGO-180 does not show any distinct peak in the region from 160 to 172 eV.

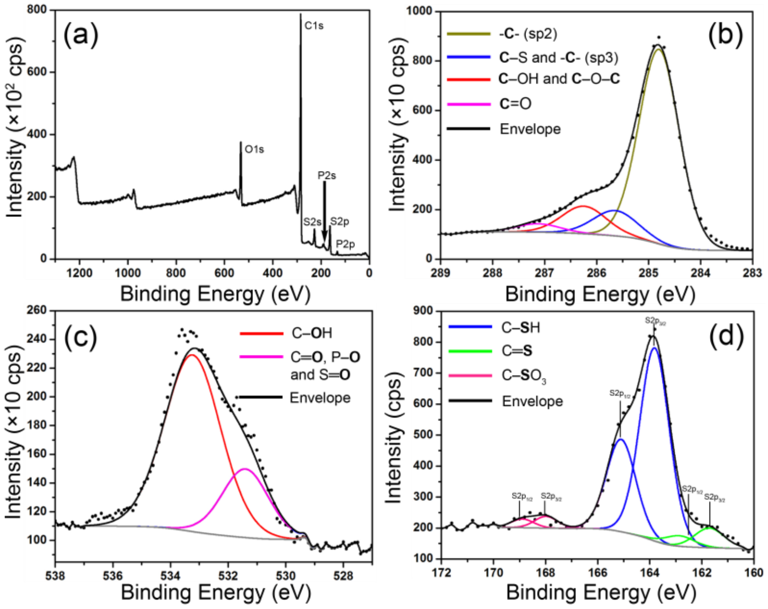

Figure S1. XPS spectra of mRGO-120: (a) wide scan; (b) high-resolution C1s; (c) high-resolution O1s and (d) high-resolution S2p

S2. Elemental Composition Analysis of mRGO-150 by XPS

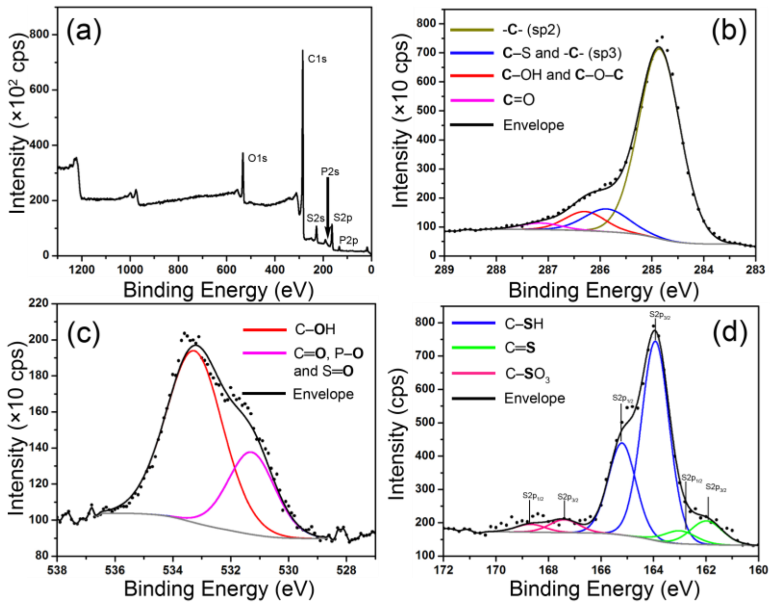

Figure S2. XPS spectra of mRGO-150: (a) wide scan; (b) high-resolution C1s; (c) high-resolution O1s and (d) high-resolution S2p

S3. Elemental Composition Analysis of tRGO-180 by XPS

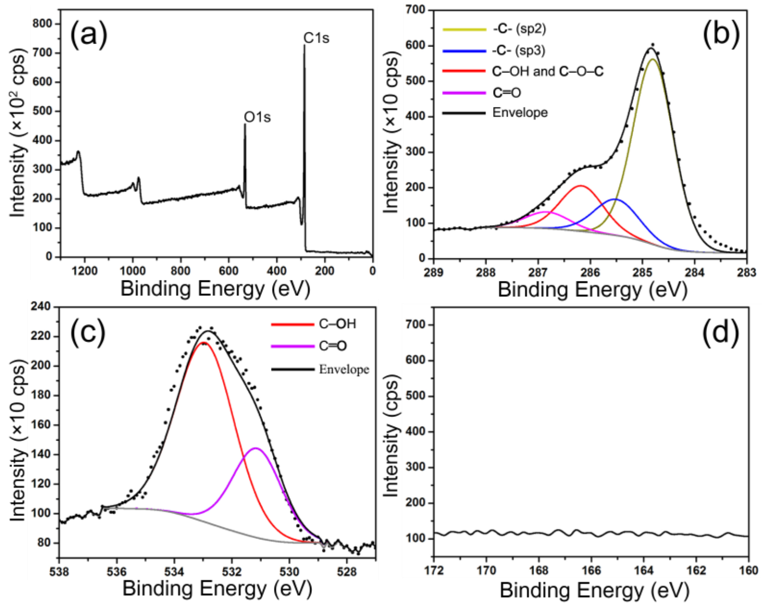

Figure S3. XPS spectra of tRGO: (a) wide scan; (b) high-resolution C1s; (c) high-resolution O1s and (d) high-resolution S2p

S4. Attenuated Total Reflectance (ATR) Fourier Transform Infrared (FT-IR) Spectra of Supernatant Solution of mRGO-180 and tRGO-180 Dispersed in Ethanol

Attenuated total reflectance-Fourier transform infrared (ATR FT-IR) was carried out with dispersion of mRGO-180 and tRGO-180 in ethanol to further study the presence of sulfur functional groups. The ATR FT-IR spectra from 800 to 550 cm^{-1} of mRGO-180 and tRGO-180 were shown in **Figure S4**. The ATR FT-IR spectrum of the mRGO-180 dispersed in ethanol clearly showed an absorption peak centered at 665 cm^{-1} which was attributed to C–S stretching band in thiol,[1] whereas the ATR FT-IR spectrum of the tRGO-180 dispersed in ethanol showed no distinct peak within the spectrum range. These ATR FT-IR spectra indicate that only mRGO contains thiol functional groups and sulfur atom in thiol functional groups attached to graphene matrix.

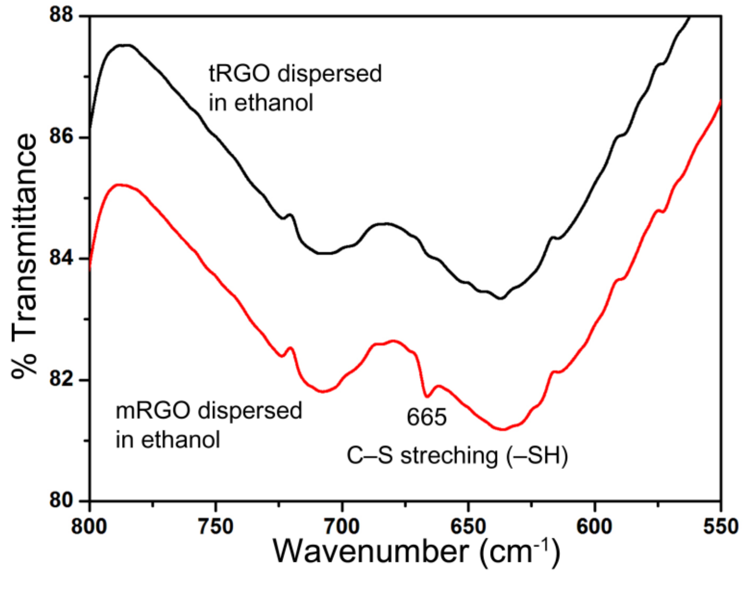

Figure S4. ATR FT-IR spectra of supernatant solution of mRGO-180 (red) and tRGO-180 (black) dispersed in ethanol.

S5. Scanning Electron Microscopic (SEM) Images of mRGO-180 powder

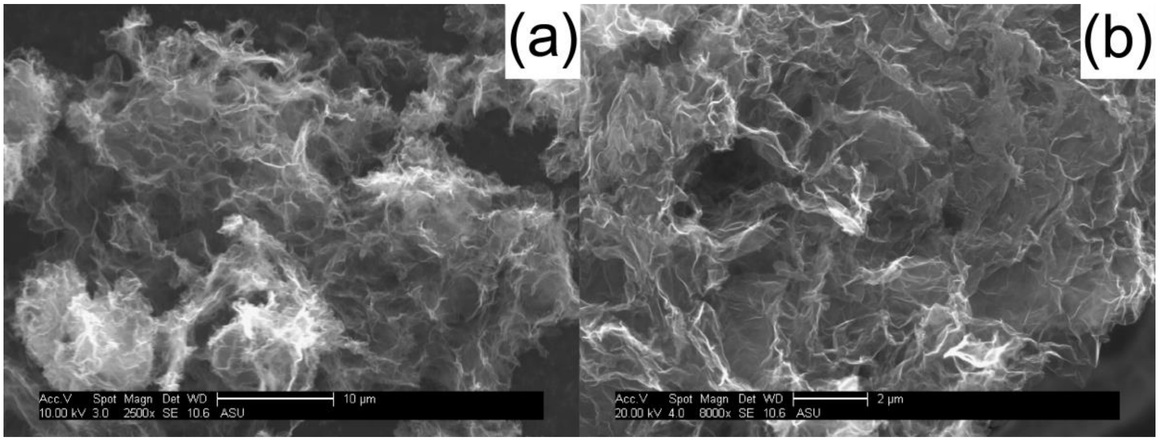

Figure S5. SEM images of the freeze-dried mRGOs: (a) low magnification and (b) high magnification.

S6. Composition Analysis of mRGO-180 using STEM Equipped with EDS

Scanning transmission electron microscope equipped with energy dispersive X-ray spectroscopy (STEM-EDS) was carried out to estimate atomic composition and elemental distribution on mRGO sheets. The dark field STEM image and the estimated atomic ratios are shown in **Figure S6**. More than 10 different mRGO sheets were investigated to estimate average O:S atomic ratio, resulting in 1: 0.9±2, which is in a good agreement with the XPS result. For STEM-EDS analysis, the estimated carbon atomic ratio was not properly quantified due to the presence of carbon grid. In STEM-EDS spectrum, Cu peak was also shown because of TEM Cu grid. The elemental mapping image of the mRGO-180 shown in **Figure S6** directly indicates that oxygen and sulfur elements were well distributed on the mRGO sheet.

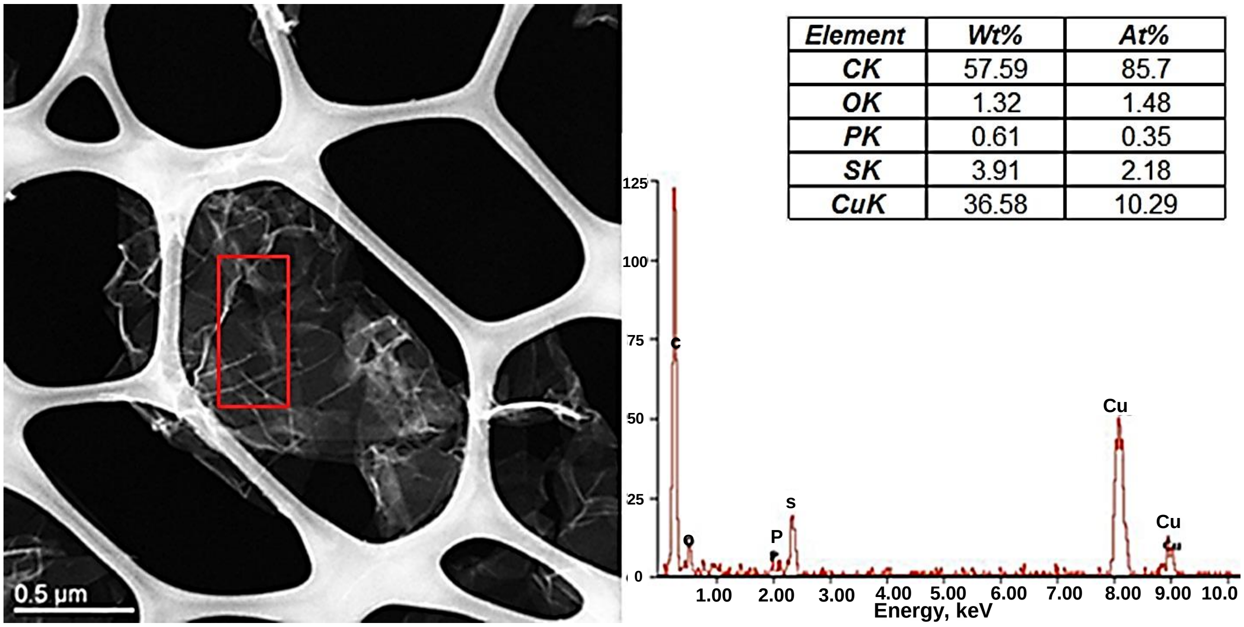

Element	Wt%	At%
CK	57.59	85.7
OK	1.32	1.48
PK	0.61	0.35
SK	3.91	2.18
CuK	36.58	10.29

Figure S6. Dark field STEM image (left), EDS spectrum and elemental composition (right) of the mRGO-180.

S7. UV-Vis Spectra of mRGO-180, tRGO-180 and GO

The reduced nature of the mRGOs could be supplementary verified by using UV-Vis spectra of the samples dispersed in ethanol (**Figure S7**). The absorption peaks of the GOs were shown around 229 nm as well as a shoulder around 305 nm which attributed to $\pi \rightarrow \pi^*$ transition of aromatic C–C bond and $n \rightarrow \pi^*$ transition of C═O.[2] The absorption peak position of the $\pi \rightarrow \pi^*$ transition was red-shifted from 229 nm for GO to 260 nm for tRGO-180 and to 273 nm for mRGO-180 due to extended aromatic C–C bond, suggesting that electronic conjugation within graphene sheet was restored.[2-3] It is important to note that the maximum absorption peak of the mRGO-180 (273 nm) was further red-shifted than that of tRGO-180 (260 nm), which indicates that the optical transition gap of the mRGO-180 is smaller than that of the tRGO-180. The further decreased optical gap might be attributed to an orbital overlap of 3s and 3p orbitals of sulfur with π-orbitals of aromatic C–C bond.

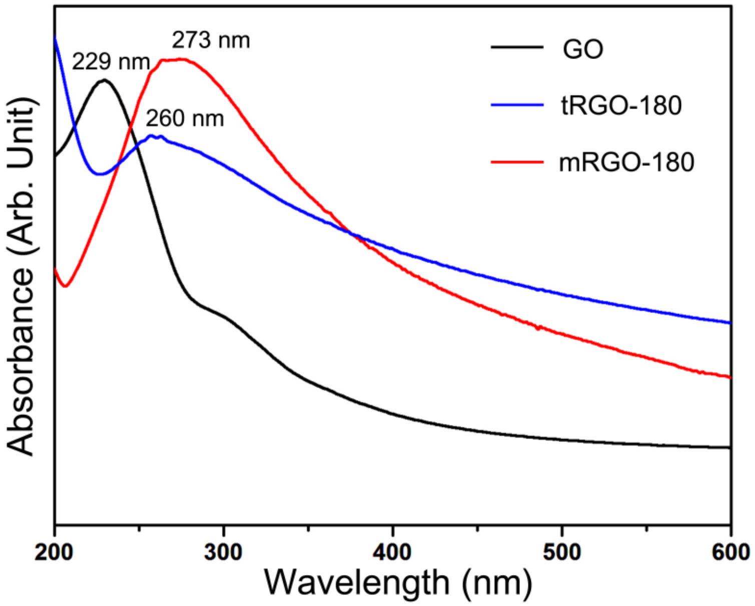

Figure S7. UV-Vis absorption spectra of mRGO-180 (red), tRGO-180 (blue) and GO (black) dispersions in ethanol.

S8. Raman Spectra of mRGO-180, tRGO-180, GO and Natural graphite flake

The I_D/I_G ratio is slightly larger (1.12) for mRGO-180 than for GO (1.08). The increase of The G band peak position in mRGO-180, tRGO-180 and GO (~1590 cm^{-1}) was higher in frequency than that of graphite. There are various factors such as varying grain size, presence of isolated double bonds,[4] etc. that can influence G band position of graphitic materials, which has been debated so far. The mRGO-180 demonstrated higher I_D/I_G ratio (1.12) than that (1.08) of the GO, which is in agreement with previous reports.[5]

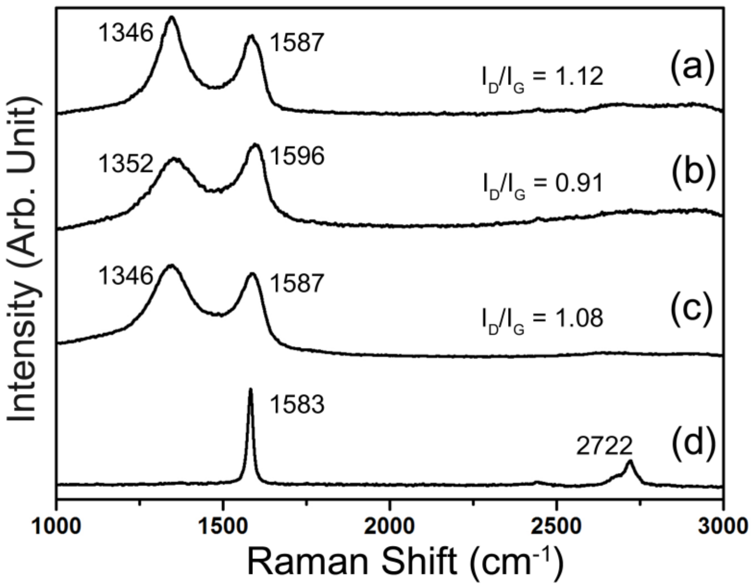

Figure S8. Raman spectra of (a) mRGO-180, (b) tRGO-180, (c) GO and (d) natural graphite flake.

S9. Estimation of Optical Band Gap of GO, mRGO-120, -150, -180 and tRGO-150: Tauc Plots

The band-to-band optical gaps for GO, mRGO-120, -150, -180 and tRG-150 were estimated to be approximate electronic band gaps, by applying Tauc's equation on their UV-Vis absorption spectra within a parabolic band approximation for amorphous carbon:[6]

$$\alpha h\nu = B(h\nu - E_{Tauc})^2$$

where α is the absorption coefficient, B is the optical constant, *h* is Planck's constant, ν is the frequency, and E_{Tauc} is the Tauc gap. In this work, the Tauc's equation is transformed into an equivalent form, $(A/\lambda)^{1/2} = \kappa(h\nu - E_{Tauc})$, which relates the measured absorbance, A, directly to the absorbed light frequency through the Beer-Lambert law, A = αbC, where b is the light path length of the analyte, and C is the concentration of the analyte. κ is a proportional constant theoretically expressed as $\kappa = (BbC/hc)^{1/2}$, where *c* is the velocity of light, but serves only as an empirical parameter here due to the simplistic nature of the Tauc model.

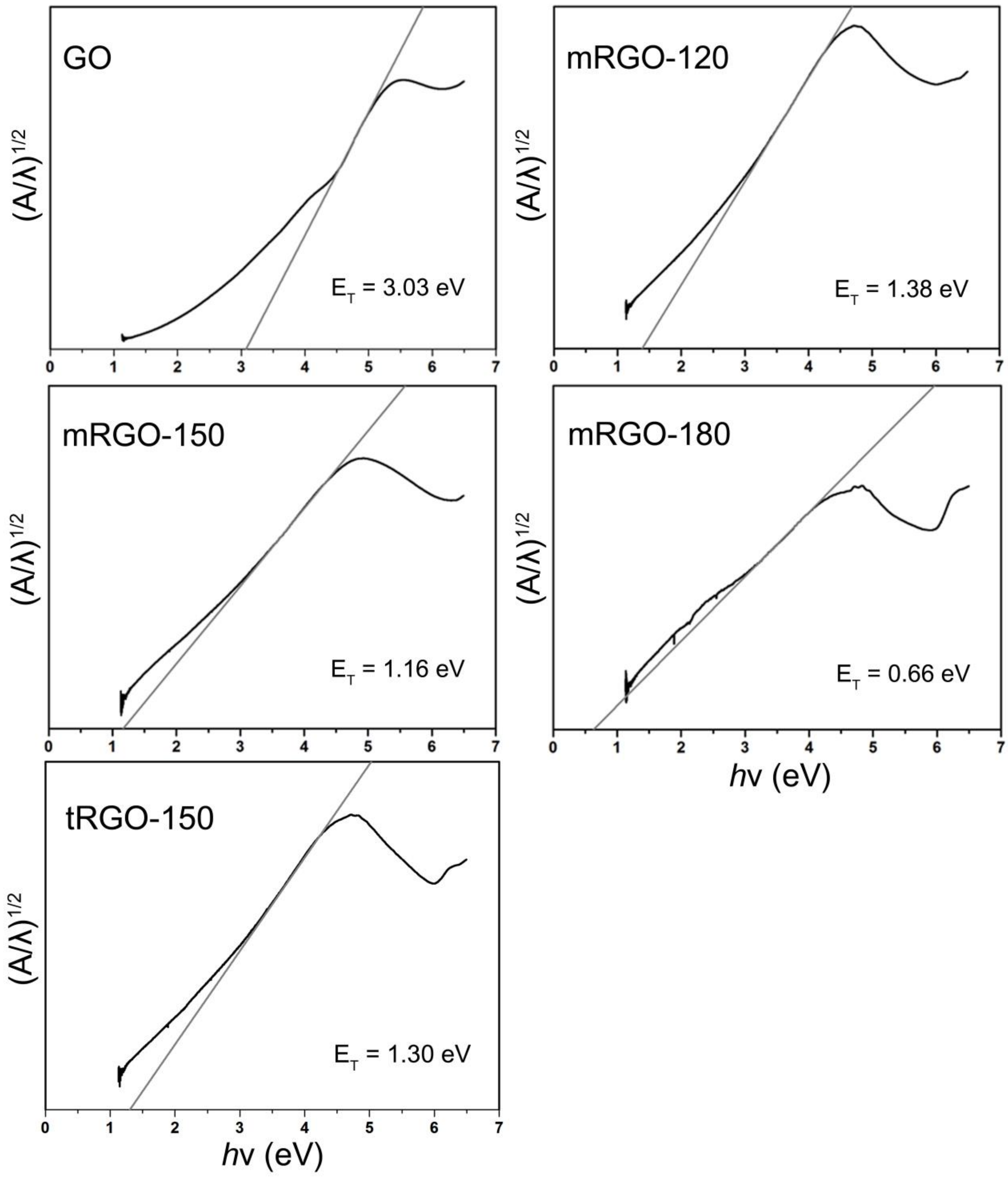

Figure S9. Tauc plots of $(A/\lambda)^{1/2}$ versus $h\nu$ for GO, mRGO-120, mRGO-150, mRGO-180 and tRGO-150.

S10. Atomic Force Microscopic (AFM) Image and Height Profile of mRGO-180 LB Film

Thickness and morphology of the individual sheet of the LB film was further investigated by atomic force microscopy (AFM) and the AFM image of the mRGO-180 LB film on freshly cleaved mica shown in **Figure S10**. The thickness of individual mRGO sheet on the mica was about 1.4 nm, indicating complete exfoliation of mRGOs in ethanol. The morphology of the sheets was similar to the sheets observed by SEM.

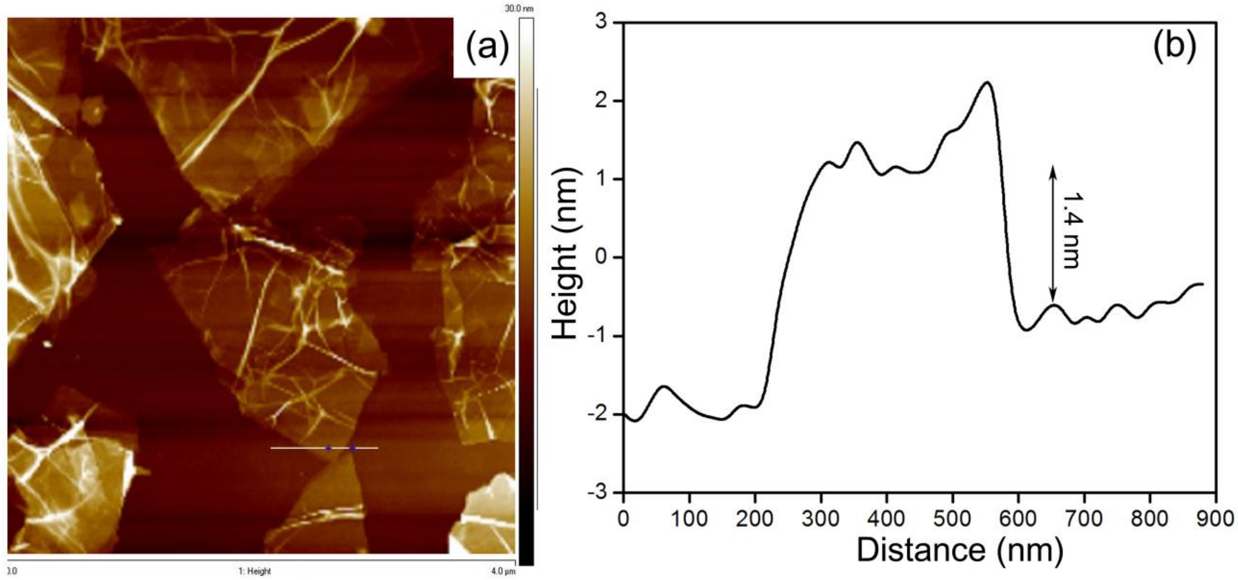

Figure S10. AFM image of (a) mRGO-180 LB film on mica and (b) corresponding height profile. The scanned area is $4 \times 4\ \mu m^2$.

S11. Optical Property Study of mRGO-180 Dispersion in Ethanol and DMF

To study optical property of mRGO, the mRGO-180 suspensions were investigated by UV-Vis spectroscopy. The mRGO-180 was dispersed in 200-proof ethanol and DMF with known concentration of mRGO, respectively and it was diluted gradually. The absorption at 660 nm of mRGO dispersions was obtained and the absorption was plotted versus concentration of mRGO (**Figure S11**), showing Beer-Lambert behavior for both ethanol and DMF solvents. The estimated absorption coefficient of mRGO-180 in those solvents is 30700 (ethanol) and 40800 (DMF) L g^{-1} m^{-1}, respectively.

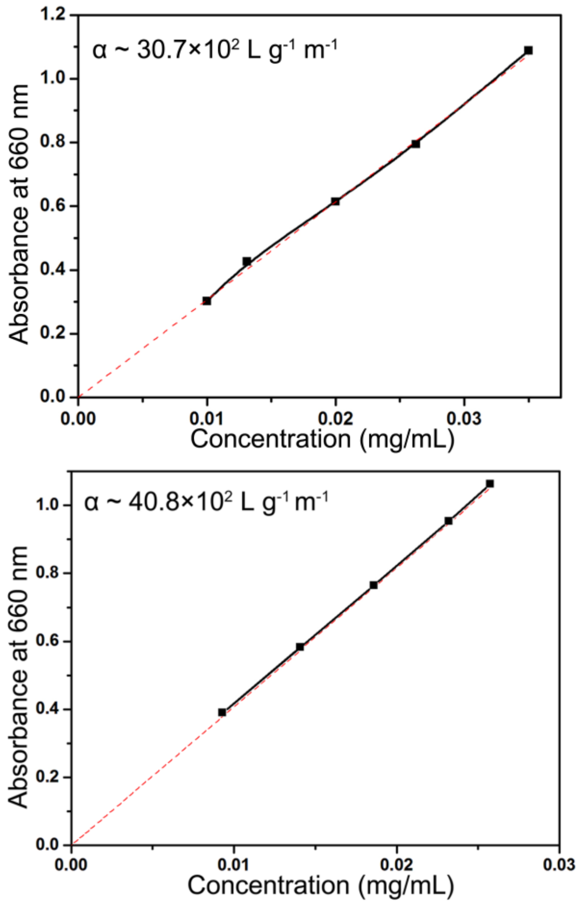

Figure S11. Absorbance at 660 nm with different concentration of mRGO-180 dispersed in ethanol (up) and in DMF (bottom), respectively.

S12. Room Temperature Current-Voltage Characteristics of mRGO-180 LB Film

Figure S12 shows room temperature current-voltage curve of mRGO-180 LB film and tRGO-180 LB film. The mRGO and tRGO LB films show Ohmic behavior in the range of voltage from -200 to 200 mV and from -800 to 800 mV, respectively. Within the voltage range, the sheet resistance of those LB films was estimated, 440 kΩ/sq for mRGO-180 LB film and 16.3 MΩ/sq for tRGO-180 LB film with similar transmittance (~ 90% at 550 nm). This result directly suggests that sulfur functional groups in graphitic matrix help to improve electrical conductivity.

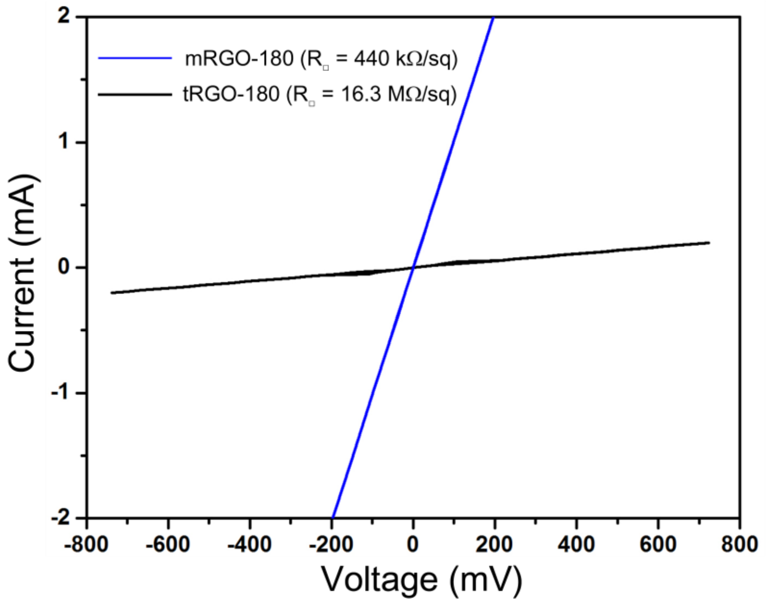

Figure S12. I-V characteristics of mRGO-180 LB film and tRGO-180 LB film at room temperature.

S13. Electronic Transport Property Study of mRGO-180 LB Film

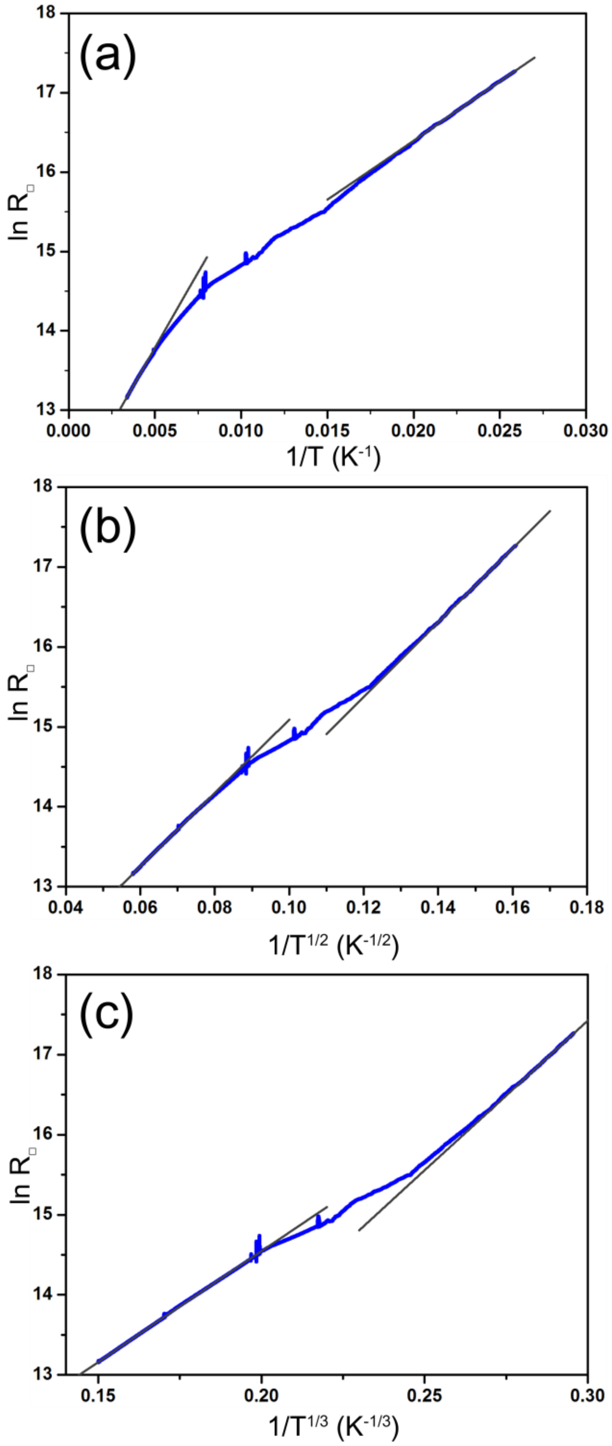

Figure S13. ln R□ versus 1/T, $1/T^{1/2}$ and $1/T^{1/3}$ for mRGO-180 LB film.

References

[1] G. Socrates *Infrared and Raman characteristic group frequencies: tables and charts,* 3rd ed.; John Wiley & Sons Ltd.: Chichester, 2001.

[2] J. Paredes, S. Villar-Rodil, A. Martinez-Alonso, J. Tascon, *Langmuir* 2008, *24,* 10560.

[3] D. Li, M. B. Müller, S. Gilje, R. B. Kaner, G. G. Wallace, *Nat. Nanotechnol.* 2008, *3,* 101.

[4] A. C. Ferrari, J. Robertson, *Phys. Rev. B* 2000, *61,* 14095.

[5] (a) S. Park, J. An, I. Jung, R. D. Piner, S. J. An, X. Li, A. Velamakanni, R. S. Ruoff, *Nano Lett.* 2009, *9,* 1593. (b) V. C. Tung, M. J. Allen, Y. Yang, R. B. Kaner, *Nat. Nanotechnol.* 2009, *4*, 25.

[6] (a) J. Tauc, R. Grigorovici, A. Vancu, *Phys. Status Solidi B* 1966, *15,* 627. (b) J. Tauc, *Mater. Res. Bull.* 1968, *3,* 37. (c) G. Fanchini, S. Ray, A. Tagliaferro, *Diamond Relat. Mater.* 2003, *12,* 891. (d) S. R. P. Silva *Properties of amorphous carbon;* INSPEC, The Institution of Electrical Engineers, London: 2003. (e) J. Robertson, S. K. Leatherhead, *Adv. Phys.* 2011, *60,* 87.
